# Pulmonary Nocardiosis: Review of Cases and an Update

**DOI:** 10.1155/2016/7494202

**Published:** 2016-03-29

**Authors:** Malini Shariff, Jayanthi Gunasekaran

**Affiliations:** Department of Microbiology, Vallabhbhai Patel Chest Institute, University of Delhi, Delhi 110007, India

## Abstract

*Nocardia*, a branching, filamentous bacteria, is widely distributed in the environment and can cause human infection in immune-compromised hosts. Inhalation of* Nocardia* leads to pulmonary disease. Microbiology laboratory processed the clinical samples from patients with respiratory infections. Smears were prepared from the samples and were stained and cultured. Five cases were positive for* Nocardia*. They were treated with the trimethoprim-sulfamethoxazole combination. The disease was cured in three patients, and two died due to other comorbid conditions leading to complications. Nocardiosis is encountered in parts of the world even where it is not endemic due to increased world travel. So physicians and laboratory staff should be aware of this and try to diagnose it. Early detection can lead to the prompt initiation of treatment and reduced mortality in these patients. Patients with disseminated or severe nocardiosis should be treated with combination therapy with two or more active agents.

## 1. Case Presentation

In the present study, five cases of pulmonary nocardiosis (PN), four males and one female, were encountered among patients attending Vallabhbhai Patel Chest Institute, a tertiary care respiratory diseases hospital in Delhi, India. They were admitted with complaints of breathlessness and increased cough with sputum production from a week to 3-month duration. They all had fever and weight loss. All were immunocompromised with four of them having the chronic obstructive pulmonary disease (COPD) with tuberculosis and one with COPD and diabetes mellitus. Sputum samples from four and bronchial alveolar lavage, bronchial aspirate, and sputum from one case showed Gram-positive filamentous branching rods with beaded appearance on Gram's staining and acid fast branching filamentous rods with beaded appearance on modified Ziehl-Neelsen staining suggestive of* Nocardia*. It was isolated on sheep blood agar from four cases. Patients were treated with trimethoprim-sulfamethoxazole (TMP-SMX) along with other antibiotics like amikacin and imipenem/meropenem. Three were discharged and advised to continue TMP-SMX for six months. Two of these were followed up and were completely free of symptoms, and their sputum was negative on smear and culture. Two of the patients died.  Table 1 shows the details of the cases.

## 2. Discussion and Update

### 2.1. Introduction

*Nocardia* is widely distributed in dust, soil, water, and vegetable matter. Inhalation of the dust particles leads to pulmonary involvement commonly caused by* N. asteroides* complex. Direct inoculation of the organism can lead to infections of the skin and subcutaneous tissue. They can disseminate from pulmonary or cutaneous focus to virtually any organ.

### 2.2. Epidemiology and Risk Factors

Nocard first described* Nocardia* in 1888 [1] which was later described by Eppinger (1890), in a man with a pulmonary disease with “pseudotuberculosis” of lungs and pleura, caseous peribronchial lymph nodes, meningitis, and multiple abscesses in the brain [2].* Nocardia* consists of more than 22 species of which* N. asteroides* complex, comprising of* N. asteroides* sensu stricto,* N. farcinica*,* N. nova,* and* N. abscessus*, is the most common. Agricultural occupation is a risk factor for pulmonary nocardiosis. Systemic immunosuppression, corticosteroid therapy, lymphoma, sarcoidosis, systemic lupus erythematosus, chronic alcoholism, diabetes mellitus, and human immunodeficiency virus (HIV) infection are other predisposing factors. Lately, it has been observed that COPD is also a risk factor for* Nocardia* infection [3].

### 2.3. Clinical Presentation

Pulmonary nocardiosis can present as acute, subacute, or chronic suppurative infection with a tendency to remit or exacerbate. PN is usually suppurative but granulomatous, or mixed variety may occur. Clinical manifestation includes pneumonia, endobronchial inflammatory masses, lung abscess, and cavitary disease with contiguous extension leading to effusion and empyema.

### 2.4. Radiological Findings

Irregular nodules, reticulonodular or diffuse pneumonic infiltrates, and pleural effusions are seen in X-ray. The progressive fibrotic disease may develop following inadequate therapy, and the diagnosis is often difficult. It can be fatal in patients with advanced HIV infection and often presents as alveolar infiltrates rather than cavitary lesion. In this situation, the X-ray findings are nonspecific and hence should be considered as a differential diagnosis of indolent pulmonary disease along with* Mycobacteria*,* Actinomyces*, and Eumycetes (*Cryptococcus neoformans* and* Aspergillus* species).

### 2.5. Laboratory Diagnosis

Demonstration of* Nocardia* in clinical sample clinches the diagnosis. Direct demonstration of* Nocardia* from sputum, bronchoalveolar lavage, bronchial aspirate, or endotracheal aspirate should be attempted.

Gram's stain smear shows Gram-positive, beaded, fine, right-angled branching filaments (<1 *μ*m diameter) which may fragment to form rods and coccoid forms of varying sizes. Most isolates of* Nocardia* are acid fast by modified Kinyoun technique that differentiates it from* Actinomyces* which is not acid fast. Silver methenamine stain is equally useful and reliable as modified Ziehl-Neelsen staining [4].

*Nocardia* spp. grow on media used for culture of bacteria, fungi, and* Mycobacteria*. Typical colonies appear after three to five days.* Nocardia* spp. appear as either buff or pigmented waxy cerebriform colonies or have a dry, chalky-white appearance with the production of aerial hyphae.

Some commercial identification systems like API 20C (Biomerieux) allow for rapid identification of* Nocardia* spp. but have the limitation of the traditional phenotypic method [5].

Molecular identification of* Nocardia* is not only quick and accurate but also helps in the recognition of new species. Various methods like ribotyping, polymerase chain reaction, restriction fragment length polymorphism analysis, and DNA sequencing are available [6].

DNA sequencing is currently the best tool for species identification of* Nocardia*. Sequencing of first 500–606 base pairs of the 5′ end of 16S rRNA gene is the recommended method [6]. All these methods have their limitations and should be used with caution.

Currently, there are no serological tests available for diagnosis of active nocardiosis due to the cross-reactivity among different* Nocardia* species,* Mycobacterium tuberculosis*,* Mycobacterium leprae,* and other Actinomycetes [7].

### 2.6. Management

Trimethoprim-sulfamethoxazole (TMP-SMX) is the mainstay of treatment and has improved the outcomes. In adults, with normal renal function and localized disease, the recommended daily dose of TMP-SMX is 5 to 10 mg/kg TMP and 25 to 50 mg/kg SMX in two to four divided doses, depending on the extent of disease. Higher initial doses (15 mg/kg TMP and 75 mg/kg SMX), given intravenously or orally, are frequently used in patients with cerebral abscesses; severe, extensive, or disseminated infection; or AIDS. Sulfonamides are the treatment of choice for disease due to* N. brasiliensis*. However, mortality with monotherapy is as high as 50% [8] especially in severely ill patients and those with cerebral involvement or disseminated nocardiosis and immune-suppression. Empirical combination therapy with amikacin and imipenem (or meropenem) or a three-drug regimen comprising of Sulphonamides, amikacin, and either a Carbapenem or third generation Cephalosporin can be used in such high-risk patients.

## 3. Conclusion

Isolation of nocardiae from sputum or blood occasionally represents colonization, transient infection, or contamination. In cases of respiratory tract colonization, Gram-stained specimens are usually negative, and cultures are only intermittently positive. Until a better tool to determine the virulence of* Nocardia* is available, the positive culture often reflects disease in immune-suppressed patients, such as patients on corticosteroid therapy, patients who undergo organ transplantation, patients with chronic lung disease, and HIV-positive patients. Therefore, PN must be suspected in patients with these risk factors and positive imaging findings. Early detection of the organism can lead to the prompt initiation of treatment and reduced mortality in these patients. Initial combination therapy with two or more active agents is recommended for patients with disseminated or severe nocardiosis.

## Additional Points

*Learning Objectives.* (i) To recognize the importance of* Nocardia* in causing lung infections. (ii) To diagnose* Nocardia* in the laboratory. (iii) To treat infections caused by* Nocardia*.

*Pre-Test.* (1) How to diagnose Pulmonary Nocardiosis? (2) How can it be treated effectively? 

*Post-Test*

*(1) How to Diagnose Pulmonary Nocardiosis?* Direct demonstration of* Nocardia* from clinical samples stained with Gram's stain and the modified acid fast stain will help in the diagnosis of Nocardiosis.* Nocardia* appears as Gram-positive filamentous branching rods with beaded appearance on Gram's staining and acid fast branching filamentous rods with beaded appearance on modified Ziehl-Neelsen staining. The diagnosis can further be confirmed by culturing the organism in solid media.

*(2) How Can It Be Treated Effectively?* Trimethoprim-sulfamethoxazole (TMP-SMX) is the mainstay of treatment. However, monotherapy may lead to treatment failures. Hence, empirical combination therapy with amikacin and imipenem (or meropenem) or a three-drug regimen comprising Sulphonamides, amikacin, and either a Carbapenem or third generation Cephalosporin can be used in high-risk patients.

## Figures and Tables

**Table 1:** Details of patients with pulmonary nocardiosis.

	Case 1	Case 2	Case 3	Case 4	Case 5
Age	76	70	57	70	42
Diagnosis	COPD with pulmonary T.B.	COPD with pulmonary T.B.	COPD with DM	Treated pulmonary T.B.	Treated pulmonary T.B.
H/o ATT	Yes	Yes	No	Yes	Yes
H/O DM	No	No	Yes	No	No
HIV	No	No	No	No	No
H/O smoking	Yes	Yes	Yes	No	No
COPD	Yes	Yes	Yes	No	No
Chief complaints	Breathlessness and increased cough with sputum for 4-5 days and loss of weight and loss of appetite	Breathlessness and cough with sputum for 20 years acutely increased for two weeks and fever for 1 week	Breathlessness and cough with sputum for 15 days	Breathlessness and cough with sputum for three months, intermittent fever for three months, and loss of weight	Persistent symptoms of fever, loss of appetite, and mucopurulent sputum for two months after ATT course
X-ray	Bilateral pneumonia	Bilateral pneumonia	—	Rt lower zone opacity	Left lower lobe collapse with consolidation
Clinical samples	Sputum	Sputum	Sputum	Sputum	BAL, BA, and sputum
SMEAR Grams	Gram positive branching filamentous rods with beads	Gram positive branching filamentous rods with beads	Gram positive branching filamentous rods with beads	Gram positive branching filamentous rods with beads	Gram positive branching filamentous rods with beads
Modified acid fast staining	Acid fast branching filamentous rods with beads	Acid fast branching filamentous rods with beads	Acid fast branching filamentous rods with beads	Acid fast branching filamentous rods with beads	Acid fast branching filamentous rods with beads
Culture on sheep blood agar	Positive after 48 hrs of incubation	Positive after 48 hrs of incubation	Culture-negative after seven days of incubation	Positive after three days of incubation	Positive after 48 hrs of incubation
Treatment	T. Septran 4 days Inj Amikacin 4 days Inj Imipenem 4 days	T. TMP-SMX 20 days Inj Amikacin 2 wks Inj Meropenem 10 days	T. TMP-SMX 5 days Inj Amikacin 5 days T. Voriconazole 5 days	Inj Amikacin 2 weeks T. TMP-SMX 4 months	Inj Amikacin 2 weeks T. TMP-SMX 4 months
Outcome	Patient expired after six days of admission	Discharged with advice to continue TMP-SMX for six months and to come for follow-up	Patient expired after six days of admission	Sputum negative after a week. Discharged with advice to continue TMP-SMX for six months and to come for follow-up	Sputum was negative after one wk of treatment. Discharged with advice to continue TMP-SMX for six mths and to come for follow-up
Follow-up	—	The patient was lost to follow-up	—	Sputum samples taken at one and two months of follow-up were negative. Complete resolution of the lesion at four months of treatment with TMP-SMX and no complaints	Smear-negative after seven days and after two weeks follow-up

COPD: chronic obstructive pulmonary disease.

ATT: antituberculous treatment.

DM: diabetes mellitus.

TMP-SMX: trimethoprim-sulfamethoxazole.

T.B.: tuberculosis.

## References

[1] Nocard M. E. (1888). Note sur la maladie des boeufs de la guadeloupe connue sous le nom de farcin. *Annales de l'Institut Pasteur*.

[2] Eppinger H. (1890). Ueber eine neue pathogenic Cladothrix und eine durch sie hervorgerufene Pseudotuberculosis. *Wiener Klinische Wochenschrift*.

[3] Emmons C. W., Binford C. H., Utz J. P., Kwon-Chung K. J. (1977). *Medical Mycology*.

[4] Garcia-Bellmunt L., Sibila O., Solanes I., Sanchez-Reus F., Plaza V. (2012). Pulmonary nocardiosis in patients with COPD: characteristics and Prognostic Factors. *Archivos de Bronconeumologia*.

[5] Mathur S., Sood R., Aron M., Iyer V. K., Verma K. (2005). Cytologic diagnosis of pulmonary nocardiosis: a report of 3 cases. *Acta Cytologica*.

[6] Brown-Elliott B. A., Brown J. M., Conville P. S., Wallace R. J. (2006). Clinical and laboratory features of the *Nocardia* spp. based on current molecular taxonomy. *Clinical Microbiology Reviews*.

[7] Mcneil M. M., Brown J. M. (1994). The medically important aerobic actinomycetes: epidemiology and microbiology. *Clinical Microbiology Reviews*.

[8] Wallace R. J., Septimus E. J., Williams T. W. (1982). Use of trimethoprim-sulfamethoxazole for treatment of infections due to Nocardia. *Reviews of Infectious Diseases*.

